# Mobile Wellness in the Workplace: Addressing the Global Men’s Healthcare Gap, from a World Health Organization Framework Perspective

**DOI:** 10.1007/s44197-024-00334-1

**Published:** 2024-12-03

**Authors:** Stephanie Davis, Wole Ameyan, Amy Medley, Carlos Toledo

**Affiliations:** 1 HIV Prevention Branch, Division of Global HIV and Tuberculosis, U.S. Centers for Disease Control and Prevention, Atlanta, GA USA; 2 Global HIV, Hepatitis and Sexually Transmitted Infections Programmes, World Health Organization, Geneva, Switzerland

**Keywords:** HIV, HIV prevention, Men’s health, Equity, Engagement

After decades of justified focus on women as the general population members most at risk for HIV, global health stakeholders are grappling with the undesired consequences that the comparatively lower focus on men has had on HIV-related outcomes and healthcare systems. As the World Health Organization’s (WHO) pivotal 2023 framework on *Men and HIV: Evidence-based Approaches and Interventions* (“the Framework”) explains, men experience increased risk of morbidity and mortality across all ten major broad disease categories by disability-adjusted life year burden [1, 2] and continue to lag behind women regarding HIV services and other health outcomes. Globally, men represent the majority of new HIV infections and lag behind women in every step of the HIV care cascade; and among people living with HIV, men have far higher risk of death from tuberculosis and from all AIDS-related causes combined than women [1].

These disparities also have an impact beyond men: recent models show that improving men’s HIV testing and treatment coverage in Africa could reduce HIV incidence among women by half [3]. Yet men have far fewer routine contact points with the healthcare system over their lifetimes than women [4], and a study quantifying time spent accessing health care services in Malawi found a sixfold difference favoring women [5]. While gender gaps in overall health do not always favor women - men can also have greater resources and higher likelihood of seeking care for known illness in some settings [6], and the burden of disability is higher for women [7] - these gaps in HIV are stark and problematic.

In this issue, Nyangulu et al. [8] report qualitative findings from implementing a WHO-recommended strategy [9] for closing the gender gap in key health service utilization: men’s mobile workplace health service. Workplace health services address two major barriers to healthcare engagement for men identified by the *Framework*– transportation and scheduling– while also providing a more welcoming environment to men than traditional clinics. The authors’ service package included HIV testing; malaria, diabetes, hypertension, and obesity screening; lifestyle counseling; and syphilis testing. By interviewing workplace managers who agreed and declined to host mobile health services, and staff who participated and declined the mobile services, the authors provide multiple perspectives on the key facilitators and barriers to using their service. These include, respectively, high-quality services on one hand, and poor promotion of the service campaign and unclear eligibility criteria on the other. Such findings support Nyangulu et al.’s key recommendations for intentional sensitization of managers on the value of workplace health services and clearer communication on eligibility. This report thus expands the body of exploratory science on potential ways to make interventions for engaging men more effective.

This contribution sits within a body of evidence that is now well-defined by global normative guidance, and ready for both implementation and practical refinements. The literature review on which the WHO *Framework* was based found substantial evidence on effective interventions to increase male uptake of WHO-recommended services in a set of key health domains: HIV and HIV/TB coinfection, STIs, viral hepatitis, and mental health and non-communicable diseases. The intended scope of the Framework is global: it “…draws evidence largely from southern and eastern Africa as that is where the vast majority of evidence has been generated, but guidance is likely applicable to diverse geographic settings across the globe.” ( [1], pg. 5). The health systems it addresses therefore vary widely in organization and engagement points, but in the sub-Saharan African context, it is common for decentralized dispensaries and local clinics to serve as the primary sources for care and screening, with or without user fees which may include sliding scales.

Qualitatively, the *Framework* identifies three interlocking factors emerging from the intervention review as key underlying factors to men’s health outcomes: *gendered health systems*,* economic vulnerability*, and *harmful gender norms*. *Gendered health systems* reflect “a common belief that clinics are not meant for men…deterring men from accessing health services” in multiple ways: “men lack routine service entry points for HIV and other services, face rigid service hours that conflict with work schedules and often must seek services in physical spaces that are primarily occupied by women.” *Economic vulnerability*, through the distinctive ways in which it affects men, creates unique logistical barriers to accessing care, “as men must spend extended periods of time searching for (or performing) income-generation activities”. *Harmful gender norms* “act as a barrier…if men believe that using services will result in loss of dignity and/or reputation in the community, or loss of ability to have new sexual partners [through inadvertent disclosure of HIV status]…and impact how health systems and health care providers interact with men as clients…when they do not provide services or health literacy campaigns that target men, and…expect and require women to bear the burden of being the ‘caregiver’ of the family, [which] affirms perceptions of clinical settings as ‘female spaces’.”

To address these barriers, the *Framework* maps solutions to male-specific barriers ( [1], p. 10). guided by three overarching strategies for person-centered services to reduce barriers for men: *easy access to services*, *quality and discrimination-free services*, and *supportive services*; and four principles: *inclusive* (across backgrounds and risk factors), *health systems-focused* (placing responsibility for engagement on health system design, not on individual men), *sustainable and scalable* (integrating multiple services and using existing health systems), and *context-specific* (informed by local data and community feedback.) Table 1 provides a summary of the *Framework’s* barriers and solutions to men’s use of HIV and related services, by person-centered care domain. These use concept-level language synthesized in part from the case studies of solutions cited in the Framework; readers interested in more specificity to inform replication may find the bibliography useful.

**Table 1:** Summary of *Framework’s* barriers and solutions to men’s use of HIV and related services, by person-centered care domain

Person-centered care domain and related male-specific barriers	Solution
**Easy Access**	
*Lack of service entry points*, due to fewer routine expected visits over the life course, like antenatal care, or ANC	Maximize use of unscheduled entry points (outpatient, emergency room, etc.) to link men to broader health services; increase male use of existing *routine entry points* (e.g., partner ANC)
*Facility services’ wait times and cost* can exclude men with travel or work conflicts	Add *community-centered services* where men work and live
*Inflexible services* (long wait times, limited hours, disease-specific facilities) also exclude men with work conflicts and can cause inadvertent HIV disclosure by men being seen at facility	Offer *flexible and efficient facility-based services*, i.e., differentiated service delivery strategies (such as self-testing, reducing wait times and/or number of visits needed)
**Quality services**	
*Negative interactions with healthcare workers* (e.g. not understanding of delays in care- seeking due to work conflicts)	Provide *positive interactions with healthcare workers* trained to deliver responsive services with kindness and respect
*Siloed services* (one visit addresses only one disease)	Offer *integrated services* screening/treating for multiple issues including at unscheduled (emergency) visits
**Supportive services**	
*Limited health knowledge* among men including how to navigate healthcare	Offer *comprehensive counseling* addressing men’s concerns (e.g., not missing work), and peer support/mentorship for *facility navigation*
*Lack of male-to-male support* especially for HIV and related conditions	Offer *peer services* and support
*Mobility and inconsistent facility attendance* especially due to work demands	Offer *virtual interventions* (e.g., telehealth and SMS-based services)

This collected evidence is ample; the systematic review for the *Framework* identified over 80 publications on barriers and potential solutions. The largest gaps remaining now are in implementation. Sharing experiences optimizing the models for delivering these services, as Nyangulu et al. do here, is therefore critical. One particular question is how integrated multi-disease interventions (see Table 1) can best be delivered to diverse populations of men in ways that are sustainable and cost-effective. For example, it is true that integrated mobile workplace services like these have great value– they can address many barriers simultaneously, may reach men who cannot be reached at facilities at all, and could potentially reach both formal and informal workplaces. However, mobile services in general also add costs and logistical demands as compared to facility-based services. Sustainable models for them (for example, through public-private partnerships, or cost-sharing with appropriate clients) are badly needed. Also needed is rigorous monitoring and sharing of the actual uptake and impact of these male-friendly models on men’s healthcare engagement across a range of settings. However, these data can be generated in the process of implementing these programmatic approaches, to close the healthcare and health outcomes gaps for men.

## Data Availability

No datasets were generated or analysed during the current study.
